# The use of telehealth in the provision of after-hours palliative care services in rural and remote Australia: A scoping review

**DOI:** 10.1371/journal.pone.0274861

**Published:** 2022-09-26

**Authors:** Pathmavathy Namasivayam, Dung T. Bui, Christine Low, Tony Barnett, Heather Bridgman, Pauline Marsh, Simone Lee

**Affiliations:** 1 School of Nursing, University of Tasmania, Hobart, Tasmania, Australia; 2 School of Health Sciences, University of Tasmania, Launceston, Tasmania, Australia; University of Leeds, UNITED KINGDOM

## Abstract

**Background:**

Accessing quality palliative care, especially at the end of life is vital in reducing physical and emotional distress and optimising quality of life. For people living in rural and remote Australia, telehealth services can be effective in providing access to after-hours palliative care.

**Objective:**

To review and map the available evidence on the use of telehealth in providing after-hours palliative care services in rural and remote Australia.

**Method:**

Scoping review using Arksey and O’Malley methodological framework. Findings are reported in accordance with the Preferred Reporting Items for Systematic reviews and Meta-Analyses extension for Scoping Reviews (PRISMA-ScR). Scopus, Web of Science, CINAHL Complete, Embase via Ovid, Emcare via Ovid, and Medline via Ovid databases were searched. Peer-reviewed studies and grey literature published in English from 2000 to May 2021 were included.

**Results:**

Twelve studies were included in the review. Four main themes were identified: 1) *Stakeholder perceptions of service*; 2) *benefits to services and users*; 3) *service challenges; and 4) recommendations for service improvement*.

**Conclusion:**

Telehealth can connect patients and families with healthcare professionals and enable patients to continue receiving care at home. However, challenges relating to patients, service, staff skills, and experience need to be overcome to ensure the success and sustainability of this service. Improved communication and care coordination, better access to patient records, and ongoing healthcare professional education are required.

**Implications:**

Protocols, comprehensive policy documents and standardized operating procedures to guide healthcare professionals to provide after-hours palliative care is needed. Ongoing education and training for staff is crucial in managing patients’ symptoms. Existing service gaps need to be explored and alternative models of after-hours palliative care need to be tested.

## Introduction

Palliative care is provided to a diverse range of people with life limiting illness who are at various stages of their illness trajectory [1]. In the contemporary Australian context, palliative care is “person and family-centred care provided for a person with an active, progressive, advanced disease, who has little or no prospect of cure and who is expected to die, and for whom the primary goal is to optimise the quality of life” [1]. Palliative care focuses on the physical, cultural, spiritual and emotional needs of the patients as well as their carers. People with life limiting illness may require palliative care for a short period of time, intermittently or consistently. This may vary from a period of months to years [2, 3].

For many Australians, palliative care is vital at the end of life, as it reduces the physical and emotional distress of dying and optimises quality of life [4]. End of life care is provided to someone who is likely to die within days to weeks of life (considered as short term) and is rapidly approaching death. This phase of palliative care considers the terminal phase of the patient and requires increased services and support to meet the higher needs of patients and their families [1, 5, 6]. Nevertheless, palliative care provision remains one of the most significant health care challenges in Australia [7]. A gap remains between having a vision of high-quality palliative care for everyone and the reality of poor access to appropriate end of life care for many people [4].

Palliative care is provided across diverse settings and requires the involvement of patients, families, and health care providers in keeping patients home, or close to, as much as possible [7]. People living in rural and remote areas mainly want to spend their last weeks at home, or a small hospital or aged care facility close to people they know, with the support of local primary healthcare professionals. To achieve this, it is important that patients have timely access to specialised advice, equipment, and practical advice in conjunction with the local approaches to palliative care [3].

There is an inconsistent provision of palliative care across Australia which raises issues of patient access to timely care and inequity of care. In addition, an absence of a specialised palliative care centre can affect the quality end of life care provided in rural and remote Australia [7, 8]. The challenges to accessing effective palliative care after-hours is further exacerbated by poor transport, lack of home visits, poor access to allied health care services, poor internet access and health care workforce insufficiencies [9].

Over the years the Australian government has made significant investments in developing and expanding palliative care services. There has been a growing focus on using technology and home-based services to provide outreach palliative care services, in facilitating people to stay at home for as long as possible [1]. Technologies such as telehealth have the ability to connect patients and their health care providers with specialist palliative care professionals for support and advice. Furthermore, video consultations with the specialist palliative care team, facilitated and supported by the local healthcare professionals, can provide additional expertise and support [3].

Nevertheless, varying levels of satisfaction with this service in regional, rural, and remote Australia were reported. Challenges such as lack of awareness about the service, limited availability of community palliative care services to support nurses in triaging patients after-hours and reduced access to medication and equipment were identified [9].

From an international perspective, several review articles support the use of telehealth in providing palliative care at home in rural and remote communities, as it improved palliative care access [10–12]. Telehealth has been used effectively in many rural and remote areas to ensure equitable care and enabled interprofessional teams to address patients and caregivers holistic care needs in supporting patient’s choice to live at home. However, several barriers to providing quality care were identified such as communication and technical issues, which can sometimes impact patient care [10–12].

### Aim

The aim of this review is to map the available evidence on the use of telehealth in providing after-hours palliative care services in rural and remote Australia.

### Review questions

What is known about the use of telehealth in providing after-hours palliative care services in rural and remote Australia?

Sub questions are as follows:

1. What are the features of telehealth used in providing after-hours palliative care services in rural and remote Australia?
2. What are the perceptions of stakeholders (services providers and receivers of care and carers) in using telehealth to provide after-hours palliative care services in rural and remote Australia?
3. What are the benefits of after-hours palliative care services using telehealth in rural and remote Australia?
4. What are the challenges of using telehealth in after-hours palliative care services in rural and remote Australia?

## Methods

This review was conducted using the Arksey and O’Malley methodological framework [13] and is in accordance with the Joanna Briggs Institute methodology for scoping review [14]. The findings are reported in accordance with the Preferred Reporting Items for Systematic reviews and Meta-Analyses extension for Scoping Reviews (PRISMA-ScR) (See S1 Appendix) [15]. A priori protocol of this study is registered on the Open Science Framework platform (https://osf.io/4qs5j) and published elsewhere [16].

### Inclusion criteria

#### Participants

Studies that referred to service providers and receivers of after-hours palliative care services using telehealth were included in this review. Providers are people who deliver and coordinate this service for patients. Receivers are patients of all ages, gender, and culture and their caregivers/families who have used the services.

#### Concept

Studies that were included were those that explored the use of telehealth to deliver after-hours palliative care and aimed to provide accessible and effective care for people whose health condition could not wait for treatment by regular face-to-face care services. Although Palliative Care Australia refers to after-hours as outside 8 am to 6 pm weekdays, outside 8 am to 12 noon on Saturdays and all day on Sundays and public holidays [17], this review included studies that used the term after-hours (without specifying times). The selected studies included at least one variable relating to the stakeholders’ perception, benefits, or challenges of the services.

#### Context

This review considered studies in rural and remote Australia, which are all areas outside Australia’s major cities. The Modified Monash Model (MMM) was used to define the locations of regional, rural and remote areas, which classifies areas from MM1 to MM7 based on a range of indicators including proximity to health care services [18]. Locations included in this review are regional centres (MM2), large rural towns (MM3), medium rural towns (MM4), small rural towns (MM5), remote areas (MM6), and very remote areas (MM7). In this review the term rural and remote is used to refer to all areas from MM2 to MM7.

#### Types of sources

Peer reviewed studies, research reports and government reports published in government and professional association websites relating to the topic were included. Articles published between January 2000 to July 2021 in English were included, due to the increasing trend of government initiatives and funding in supporting telehealth research in Australia from the year 2000.

#### Search strategy

The following databases were searched for literature in July 2021: Medline Complete (EBSCO), CINAHL Complete (EBSCO), Scopus, Web of Science Core Collection, Embase (Ovid), PsycINFO (Ovid), and Emcare (Ovid). A full electronic search strategy for Medline Complete was developed to include all possible terms (See S2 Appendix) [19]. A targeted search of grey literature was conducted within relevant organisations’ websites such as Care Search and Palliative Care Australia. In addition, reference lists of the selected articles were scanned for additional articles.

#### Study selection

**Fig 1:**
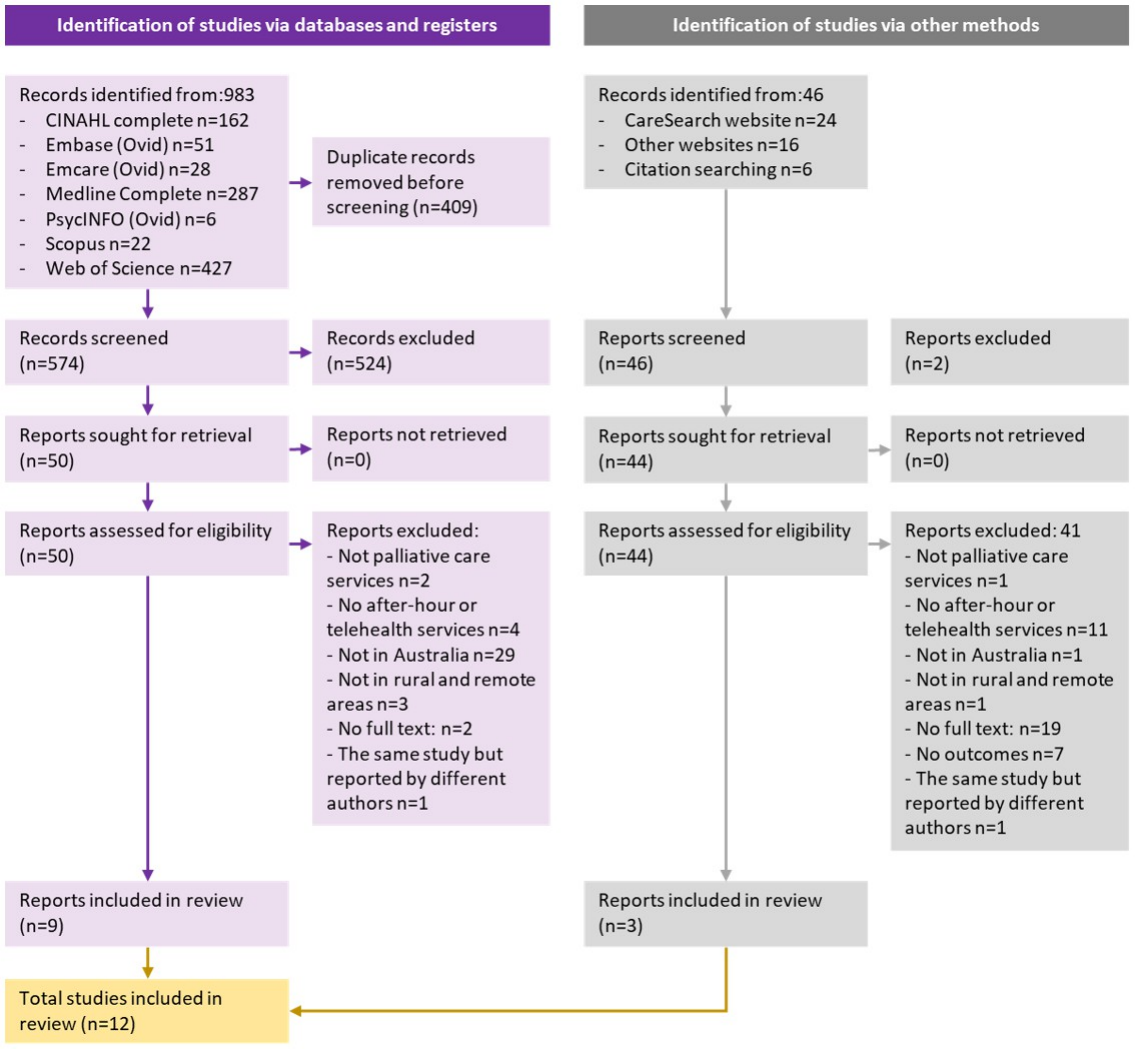
Completed PRISMA 2020 flow chart 20210903.

Two independent reviewers (DB and CL) conducted the search. The identified records were screened by two independent reviewers for eligibility using a two-stage sifting approach to review the title, abstract, and full-text articles. Disagreements between the reviewers were observed on one article (8.3%), which was resolved by involving a third reviewer (PN). The study selection process and reasons for exclusion of publications are presented using the updated Preferred Reporting Items for Systematic Reviews and Meta-analyses (PRISMA 2020) flow diagram (Fig 1) [20].

### Data extraction

An adapted data extraction tool was used to extract data (See S3 Appendix) [13]. The extracted data included specific details about the population and sample, setting, methodology, outcome measures and general findings. The data extraction tool was initially tested with the first two articles and then modified during the process of extracting data from each included paper. Disagreements raised during data extraction between the reviewers were resolved through discussion with the third reviewer.

### Data analysis

The data extracted was screened and managed using MS Excel (Microsoft Corporation, Redmond, WA, USA). Descriptive themes were developed using the first two steps of thematic synthesis techniques [21].

## Results

The literature search identified 1029 studies. After the removal of duplicates, 620 studies were screened for eligibility, of which 12 were included in the full review. An overview of the characteristics of the studies included in the review is available in S3 Appendix.

### Studies characteristics

A total of 12 Australian studies were reviewed: five were conducted in Victoria [22–26], three in New South Wales [27–29], two in Queensland [30, 31], one in Tasmania [32], and one study was conducted nationally [33]. The research methods used in this study were quantitative methods [22, 29–33], mixed methods [25–28], and qualitative methods [23, 24]. The quantitative studies’ methods, were descriptive [22, 29, 33], retrospective [30–32], and non-randomized trial [30]. The participants in the studies were palliative care patients and their carers [22, 25, 27, 28, 30–32], as well as service providers who included General Practitioners (GPs), nurses, and managers [24, 29, 33].

### Service information

Seven studies exclusively reported on services provided afterhours [22–24, 26, 28, 29, 31] while the remaining five studies were on all hours coverage [25, 27, 30, 32, 33]. These services were mainly established by government health services [23–26, 28, 29, 32], followed by service authorities based in hospitals, hospices, or health centres [22, 27, 30, 31, 33].

The majority of telehealth services that provided after-hours palliative care services were assisted by registered nurses (n = 7) [22, 24, 26–29, 32, 33] followed by nurse specialists (n = 4) [25, 29–32], GPs, specialist physicians (n = 2) [25, 27], and others including after-hours coordinators and managers (n = 3) [22, 24, 29]. One study did not identify the service assistance type [23]. The mode of communication in providing this service were via telephone [22–24, 26–29, 31–33], Web Real Time Communication platform [25], and videotelephony package [30].

### Stakeholder perceptions of service

Health care services, patients and their carers provided positive feedback, were mainly satisfied with the use of telehealth in after-hours palliative care and highly valued the service [23–26, 28–32]. Stakeholders found the service to be simple and effective [30, 32]. Improvements in the quality of care for palliative care patients was notable, through better patient management and effective integration into community-based palliative care [25, 27]. The service provided patients and carers with security [29]. In addition, families found the service to be useful and acceptable for paediatric oncology palliative care patients [30]. Most stakeholders in the studies perceived after-hours telehealth service to be useful except for two studies [23, 33] that reported low levels of satisfaction with this service. This was due to poor access to general practitioners (GPs) or locums afterhours and difficulty obtaining emergency medication afterhours [23]. Currie et al. [33] did not identify the reason for a low uptake of the service however, noted the poor response rate to evaluation surveys as a limitation of their study [33]. Although the participants indicated some satisfaction, they revealed several gaps which were present in the service [23]. Furthermore, low uptake and low call volume affected the cost-effectiveness of one telehealth service [33].

### Benefits to users and services

The use of telehealth in providing after-hours palliative care services had several benefits to users (patients and carers) and service providers [25–30, 32]. These included cost savings for patients and their carers as the service was free for the caller and helped reduce patient travel costs and travel time [25, 29, 30]. The service also helped lessen feelings of isolation for patients and their carers especially at night. It helped carers manage the complex process of caring for someone who is dying and helped to facilitate advice and services in the absence of a GP [28]. Furthermore, after-hours telehealth services allowed patients to stay at home, and provided nurses with insights into the carers’ experiences [29]. The service enabled better patient management, maintenance, and improvement of patient performance levels [25, 26]. The delivery of palliative care afterhours was possible using remote interaction via video conference calls that enabled the establishment of patient-clinician rapport [25]. The service allowed for more equitable access to specialist health care support in rural and remote areas [30].

Service providers highlighted benefits such as reduced ambulance callouts and emergency department (ED) presentations [27, 28, 32]; more equitable access to care [30]; general practitioners and other healthcare professionals feeling supported in managing patients in the community [25, 28]; reduced patient visits by healthcare professionals [25]; early management of patients’ symptoms [25]; and integration of services for greater synergies and efficiencies [28]. In addition, health care services reported significant cost savings due to fewer call outs to ambulance services and ED presentations [25, 28, 32]; reduced use of health care resources [25]; and cost effectiveness in operating the service [28].

Although using telehealth to provide after-hours palliative care had many benefits, it presented several challenges.

### Service challenges

Service challenges identified in this review can be categorised into issues relating to service provision [23, 25, 26, 30], healthcare professionals [23, 24, 26–29], and patients [23, 24, 27, 29, 30].

#### Service provision

The identified challenges relating to service provision were lack of mobile phone coverage, reception, and connectivity [23, 25], poor uptake, difficulty in maintaining time frame and contacting other services [26]. Limited access to computers resulted in difficulty accessing resources and databases [27]. Moreover, using the same after-hours telehealth model in rural and remote areas, as in metropolitan areas can be challenging [26].

#### Healthcare professionals

Challenges relating to healthcare professionals were associated with a lack of care coordination between health care providers [23, 24, 26, 27, 29], and a lack of staff skills and experience [23, 24, 27–29]. The lack of care coordination related to a lack of communication between health care providers [26], inclusion of multidisciplinary teams in care planning, and bereavement and informal staff supports [27]. Inadequate discharge planning led to a break down in patient care coordination [24], consequently, leading to poor patient and family care [26]. Access to medication after-hours for patients were challenging [23, 24, 26]. This was due to lack of emergency medication supply in patients’ homes [23], timely anticipatory prescribing [24], and lack of medication availability after-hours [26]. Healthcare professionals providing the telehealth service were at times unfamiliar with the patients and their care [27]. Additionally, some health care services lacked awareness about the after-hours telehealth service [29].

The lack of staff skills and experience was related to a shortage of trained staff [26], and a lack of staff confidence and education [27, 29]. Using telehealth to provide after-hours palliative care service was perceived as a necessary adjustment process for healthcare professionals [28]. Concerns about local doctors’ lack of palliative care knowledge in managing patients after-hours, lack of clinical guidelines in supporting staff in clinical decision-making during triage, and healthcare professionals not being able to meet patient/family needs were highlighted [24, 28]. Furthermore, after-hours GP access and low GP participation was problematic [23, 24, 26]. Staff changes in services caused further disruption to patient care [26]. When patients were terminally ill, the preference was often for experienced palliative care nurses for after-hours telephone advice and visits [23].

#### Patient issues

There was poor uptake of service due to the stoicism and hardiness of the rural population in Australia who regard hospitals and medical services as a last resort [26, 29]. There was also a lack of awareness of the number to call and carer’s reluctance to use the service as they did not want to disturb staff and preferred calling the ambulance service as they perceived it to be an emergency [27]. Some patients preferred to wait for the nurse to visit the next morning, which at times resulted in crisis situations requiring patients to be transferred to emergency departments [23]. The length of time some patients were enrolled in the service was short [24] and others died before the videotelephone links could be established [30].

### Recommendations for service improvement

Recommendations for service improvement highlighted in studies were related to healthcare professional education [23, 26, 29]; improved communication and care coordination [23, 24, 26, 27]; better access to patient records [24–27]; and service promotion and publicity [26, 28, 29, 33].

#### Healthcare professional education

It is important for triage nurses and palliative care service providers to have ongoing education and training, which also needs to be updated regularly [23, 26, 29]. Clinical skills, education on symptom management, and knowledge of local resources were all critical to managing patients’ symptoms [23, 28].

#### Improved communication and care coordination

There is a great need for communication and care coordination to be improved for patients and their families. Patients and their families needed to be reassured that after-hours support was available to them [23]. Patients and families at times were more comfortable knowing that familiar staff were providing the after-hours service as this allowed for continuity of care [27]. For Aboriginal communities in particular, it was important to be able to talk to familiar people [24].

Improved care coordination between services had great benefit to patients and was important in reducing ED after-hours admissions [24, 26–28]. Effective communication between the triage nurse and palliative care providers, regular case conferences, and shared service protocols enabled improved coordination of patient care [26, 27]. However, role delineation and the central point of contact needed to be clear [24, 26]. Services can be further improved by having a clear goal of care for patients, advanced care planning, patients and carers having a copy of the care plan and regular family meetings [24]. Protocols, comprehensive policy documents and standardized operating procedures could further guide healthcare professionals to provide patient advice [28]. Policies, procedures and documents to support decision making relating to patient care should be clear, accessible and made available to healthcare professionals [24]. Maximising the palliative care team capacity and ensuring adequate staffing in improving care coordination was important [26, 28]. Moreover, the availability of palliative care specialists after-hours was important in supporting healthcare professionals to provide care using telehealth [24].

#### Better access to patient records

Collaboration and information sharing between health care providers needs to be improved with a central contact point for accessing patient information [26, 29]. Access to electronic patient records should be made available to healthcare professionals [27]. This can be possible through implementation of computer information systems which consist of a compatible state-wide software [25–27]. Furthermore, software compatibility between services, good service management and accreditation was also considered important [25].

#### Service promotion and publicity

Service promotion was needed to improve patient and carer awareness of service and increase uptake [26, 33]. Giving patients and their carers visual prompts, such as fridge magnets, with service contact details further enhanced promotion of the service [28, 29, 33].

## Discussion

The use of telehealth to provide after-hours palliative care, generally increased and improved access to care for patients and their families in rural and remote Australia. This service provided timely access to care further reducing feelings of isolation for patients and their families and creating a sense of security especially at night. Many patients were connected quickly with care providers and were able to connect remotely with palliative care experts who might otherwise be difficult to access due to the challenges of living in a remote area. The use of technology was able to overcome time and geographical barriers [11, 34].

There were significant cost savings for patients as telehealth reduced patient travel costs, travel times and was free for the caller. Patients living considerable distances from their nearest palliative care provider and whose condition limited their ease of travel were able to access palliative care after-hours. This allowed patients to continue to remain in comfort at home Furthermore, technology enabled remote consultations between primary providers and palliative care specialists [34–36].

Providing exclusive in-person service in rural and remote areas can pose significant staffing challenges. Through telehealth services, care was provided to many more patients compared to the focus being solely on home visits [37]. Patients were able to develop close connections and trusting relationships with healthcare professionals, as consultation was conducted in their home environment [38]. This provided patients with a secure environment in which they felt comfortable discussing their personal feelings and needs. This finding however contrasted with Bonsignore et al. [37] who noted that telehealth service was unable to replace the depth of in-person, face to face care.

Similar to the findings of this scoping review, Steindal et al. [11] and Gordon et al. [12] found telehealth to be feasible for use in palliative care for remote communities. It reportedly reduced visits by clinicians [12]. In addition, the software used in telehealth needs to be simple, effective, reliable, and safe with the highest level of security for confidentiality [39].

Although healthcare professionals and patients consider telehealth to be more acceptable for younger patients, very few issues relating to usability of the equipment for telehealth were reported among service users. Older adults became increasingly competent in using the technology over time [38]. However, the current scoping review reported that in remote areas mobile phone coverage, reception and connectivity can be an issue. In addition, older people and digitally inexperienced patients needed to be supported to build their level of confidence and preparedness to engage with virtual care [39]. Nevertheless, the success and sustainability of telehealth are dependent on factors such as reliable and secure equipment, connectivity and clinician support, and flexibility [36].

There was a great need for the service to be promoted among patients and carers in raising awareness and improving uptake of service. Some patients used the service for a limited time due to their illness or delay in establishment of service. The lack of or late referral or initiation of care was often a key barrier to providing effective palliative care for under-served populations [4]. Gordon et al. [12] highlighted the need for stakeholder buy-in before launching telehealth service, adequate preparation, and additional budget to cover training.

The use of telehealth to provide after-hours palliative care was largely effective in managing patients’ physical and emotional symptoms. It enabled carers to manage the complex process of caring for someone who is dying at home. Patients reported increased quality of life, and positive mood changes [12]. It provided patients an opportunity to self-manage their illness and symptoms through feedback from telehealth services. This enabled patients to understand their conditions and develop self-management techniques through the facilitation of patient autonomy [38]. In contrast, there were contradicting results on whether telehealth services improved burdensome symptoms and quality of life, with some patients experiencing higher symptom burden and others reporting an improved quality of life [11]. Further, telehealth was not an appropriate service for discussing serious diagnosis or end of life issues as it lacked necessary interpersonal dynamics [11].

This scoping review highlighted several challenges with anticipatory care planning. Inadequate discharge planning led to a breakdown in patient care coordination, consequently leading to poor patient and family care. Furthermore, a lack of emergency medication availability after-hours and timely anticipatory prescribing was a limitation to patients receiving appropriate care. Effective, targeted and culturally sensitive anticipatory care planning, would most likely increase the likelihood for people being cared for and dying at their place of choice [40]. It is crucial that telehealth services providing after-hours palliative care include an interprofessional team, to effectively address the holistic needs of patients and their caregivers. The interprofessional team would be dependent on the patient’s diagnosis and the family’s needs [12]. However, lack of palliative care knowledge in managing patients’ complex symptoms after-hours can cause challenges, and lead to unnecessary patient and family distress.

Collaboration and information sharing after-hours was important to improve patient care. Patient’s record needs to be made available electronically for timely access of patient information. This can be achieved through computer information system. Electronic information sharing supported interdisciplinary collaboration and communication in networks. This helped address the limited availability of palliative care expertise and enabling timely expert consult [34]. However, not being able to look through the same documents in real time can be challenging when discussing issues like patient’s medication or care directives [36].

The evidence from the review suggested that using telehealth to provide after-hours palliative care can address some of the issues of access for patients and families living in rural and remote Australia. However, there is a great need for this support service to be integrated from the time of diagnosis, through outpatient care and support, and palliative care rather than for palliative care in isolation [30]. This will help ensure continuity of care for patients and their carers. Mechanisms of interfacing with larger-scale telephone support call centers need to be investigated to explore the difference between generic and disease-specific approaches and assess the cultural appropriateness of this model of intervention in culturally diverse groups [28]. Additionally, high-level evidence is needed to make a convincing argument of the value of after-hours calls for underserved communities living in rural and remote Australia [28]. Further research to identify the value of after-hours service to patients and carers is required to assess the impact that the after-hours service has on dying at home [32, 33]. The existing service gaps need to be explored and alternative or modified models of after-hours care need to be tested [23]. In addition, robust evidence into the efficacy of the service remains scant, and randomized controlled trials in palliative-care settings are recommended [25].

### Limitations and strengths

The findings of this review are limited to rural and remote Australia and did not make comparisons to urban areas. Furthermore, the review was limited to how telehealth was used to provide after-hours palliative care to patients and their carers and did not explore the use of this service by generalist service providers in rural and remote areas to support patients at home. The focus of the review was on telehealth services which provided care to patients who are being cared for in a home environment and not necessarily in a residential aged care facility, where the need for palliative services is also great. Nevertheless, the review provided great insight into the strengths and limitations of the use of telehealth in providing after-hours palliative care in rural and remote Australia. The review has implications for practice, research, and policies in that it highlighted the feasibility of using telehealth to provide after-hours palliative care. It highlighted the benefits of the service as well as the challenges experienced by patient, carers and health care providers and the need for further improvement to enable better access to care.

## Conclusion

The use of telehealth in providing after-hours palliative care is generally able to overcome many of the geographical and access barriers experienced in rural and remote Australia. This is achieved by using technology to connect patients and their families with healthcare professionals. This service increases patient access to palliative care in a timely manner in managing their symptoms and reduces feelings of isolation. Furthermore, it enables patients to be continued to be cared for in their home environment. Nevertheless, to ensure that the service is efficient and sustainable, there are some challenges that need to be overcome, including poor internet coverage and connectivity, inadequate communication between healthcare professionals, and lack of care coordination across providers. The review highlights the need for protocols, comprehensive policy documents and standardized operating procedures to guide healthcare professionals to provide after-hours palliative care using technology.

In addition, ongoing education and training for triage nurses and palliative care services is needed in managing patients’ symptoms effectively.

## Supporting information

S1 Appendix PRISMA-ScR checklist. (DOCX)

S2 Appendix Search strategy. (DOCX)

S3 Appendix Study characteristics. (DOCX)
