# Supplementary material for: The use of telehealth in the provision of after-hours palliative care services in rural and remote Australia: A scoping review
Source: PLoS One. 2022 Sep 26;17(9):e0274861. doi: 10.1371/journal.pone.0274861 (PMC9512207; doi:10.1371/journal.pone.0274861)
Supplement: S2 Appendix — (DOCX) [file pone.0274861.s002.docx]

Appendix 2. Search strategy for MEDLINE Complete.

| **Search** | **Query** | **Records retrieved** |
| --- | --- | --- |
| #1 | TX telehealth OR telemedicine OR telemonitor* OR telepractice OR telenursing OR telecare OR mobile health OR mHealth OR eHealth OR e-Health OR telecare OR virtual care OR teletherapy OR telepractice OR teleconsult* OR remote consult* OR distance counsel* OR telecommunicat* OR telecommut* OR teleassist* OR teleconferenc* OR videoconferenc* OR videotelephon* OR telephone triage OR telephone assessment OR telephone advice OR Internet | 460,868 |
| #2 | TX palliative* OR end of life* OR terminal* OR hospice* OR dying OR death | 3,167,173 |
| #3 | TX after hours OR after-hours OR out of hours OR on call | 987,988 |
| #4 | TX rural OR remote OR suburban OR regional OR bush OR outback OR outskirts | 1,631,000 |
| #5 | TX Australia* | 1,247,872 |
| #6 | #1 AND #2 AND #3 AND #4 AND #5 | 6,556 |
| #7 | **Limiters** - Date of Publication: 20000101-20210531 | 6,441 |
| #8 | **Narrow by Language:**- English | 6,402 |
| #9 | **Narrow by Subject Geographic:**- Australia | 240 |

Adapted from Namasivayam P, Bui DT, Low C, Barnett T, Bridgman H, Marsh P, Lee S. 2022. Use of telehealth in the provision of after-hours palliative care services in rural and remote Australia: A scoping review protocol. PLoS One. 17(1):e0261962.
