# Supplementary material for: The use of telehealth in the provision of after-hours palliative care services in rural and remote Australia: A scoping review
Source: PLoS One. 2022 Sep 26;17(9):e0274861. doi: 10.1371/journal.pone.0274861 (PMC9512207; doi:10.1371/journal.pone.0274861)
Supplement: S3 Appendix — (DOCX) [file pone.0274861.s003.docx]

**Appendix 3. Study characteristics.**

| **No.** | **Authors (Year)** | **Aims** | **Design, sample, & locations** | **Services & duration** | **General findings** |
| --- | --- | --- | --- | --- | --- |
|  | Cromwell et al. (2003) | Describe an evaluation of the Griffith Area Palliative Care Service (GAPS) project. | Mixed methods. 95 palliative care patients. Griffith, New South Wales. | A 24/7 palliative care service. 17 months. | The GAPS project has brought a notable improvement in quality of care. |
|  | Wilkes et al (2004) | Evaluate an after-hours telephone support service for palliative care patients and their families at home. | Descriptive evaluation. 48 health care professionals using the service (GPs, Clinical Nurse Specialists, Nurse Unit Managers, Medical specialist, visiting specialist from hospitals, private practice and community agencies); 21 nurses providing the service (RNs, ENs, clinical nurse specialists, clinical nurse consultant and nurse unit managers). Grafton, New South Wales. | An after-hours telephone support service. 8 weeks. | Knowing the service was there was a great security and reduced the sense of isolation predominant in the experience of rural families caring for a palliative care patient at home. Based on the positive evaluation, the area health service provided ongoing funding for the service and is exploring avenues to extend the program into other areas |
|  | Phillips et al (2008) | Supporting patients and their caregivers after-hours at the end of life: the role of telephone support | Mixed method evaluation. 350 people registered with the service. Mid North Coast, New South Wales. | An after-hours telephone support service. 20 months. | Access to a palliative care after-hour telephone support service is highly valued, particularly by caregivers. The majority of after-hours calls occur in the time from 6 pm to midnight and caregivers value support and advice given by health professionals.  The telephone is a suitable vehicle to provide this type of health care service. |
|  | Bensink et al. (2009) | Investigate the acceptability of videotelephony as a method of service delivery for paediatric oncology palliative care. | Non-randomized trial. 11 families palliative care families (5 in city, and 6 in regional and remote). Brisbane, Queensland. | A 24-hour videotelephony service to support paediatric oncology-related palliative care in the home. 12 months. | Providing videotelephone support to paediatric oncology-related palliative care families, as part of the routine service provided, was acceptable. |
|  | Ciechomski et al. (2009) | Report the preliminary findings of a project exploring after-hours palliative care service provision in three regions of Victoria, Australia. | Thematic analysis of transcribed semi-structured interviews. GPs, Nurses, Managers, & terminally ill people and their carers. Inner urban (region 1), semi-urban and retirement area (region 2), & small and medium towns and isolated areas (region 3). Victoria. | An after-hours palliative care service. | Some examples of satisfaction with services were indicated, however, gaps in the provision of after-hours palliative care services, such as training, remuneration and availability of general practitioners, care planning that includes after-hours care, interdisciplinary team communication issues, staff safety and after-hours telephone support and information for families, were all identified by stakeholders. |
|  | Bradford et al. (2012) | Gain an understanding of the types of calls made who was calling, when and why the call was made and to determine the effectiveness of nurse-led management of after-hours calls. | Retrospective study. 73 families caring for a child with incurable cancer. Queensland. | An after-hours phone service to support children with incurable cancer & their families. 8 years. | The after-hours phone service has proven to be a simple, effective, and valuable service, which is consistently accessed by families, regardless of distance from the hospital. |
|  | Chidell et al. (2013) | Describe an after-hours palliative care telephone support service in Victoria. | Descriptive study. 101 patients in community palliative care services and their families. Eastern Metropolitan Region, Hume Region, and Barwon South-Western Region, Victoria. | An after-hours telephone triage. 2 years. | - Caritas Christi Hospice in Victoria, Australia, provides an after-hours  telephone triage (AHTT) support service to three community palliative care services in Melbourne and the surrounding area.  - Clients can contact the AHTT between roughly 5 pm and 7 am on weekdays and any time at the weekend, and the duty nurse will try to resolve any issues over the phone where possible.  - The AHTT staff have access to electronic patient record systems in two of the palliative care services and receive weekly patient information by fax from the third service. |
|  | Victoria Department of Health (2013) | The evaluation of the pilot projects, and to provide recommendations for the recurrent allocation of funds | Mixed-methods design. Stakeholders including service provider management, service provider staff, 107 palliative care clients and their carers, triage staff, project officers and project managers Area 1: Eastern Metropolitan Region, Barwon South-Western Region and Hume Region, Victoria. Area 2: Grampians Region and Loddon Mallee Regional, Victoria. | An after-hours palliative care services via telephone triage. 27 months. | The most important factor in the appropriate provision of after-hours community palliative care is the level and quality of communication. This applies both within multidisciplinary teams, and between team members, clients and their carers. GPs are an integral part of the team, and they need to be involved in forward planning that anticipates possible crisis and caters for the potential need for medication after-hours. The adequate training of triage staff and providing appropriate protocols for their use have been shown to be important. Finally, the quality of information transfer systems and of course client satisfaction are very important indicators of the quality of service provided. |
|  | Gippsland Region PC Consortium (2014) | Get updated information about after-hours palliative care practices and feedback about the proposed Gippsland model for after-hours palliative care. | Semi-structured interviews. Palliative care (PC) co-ordinator (or delegate) of 12 community PC services. Gippsland, Victoria. | An after-hour palliative care telephone triage. 2 years. | Eight of nine services prefer to continue providing the telephone triage service for palliative care clients locally. |
|  | Baird-Bower et al. (2016) | Evaluate the use of a state-wide after-hours palliative care support number. | Retrospective study. 146 patients with life-limiting illness and a life expectancy of less than 12 months. Tasmania. | A 24/7 multidisciplinary primary palliative care service for people with life-limiting illness and a life expectancy of less than 12 months. 6 months. | 24-hour palliative care telephone support was found to be a valuable tool for all individuals involved in the care of end-of-life patients. |
|  | Currie et al. (2016) | Enhance national provision of palliative care and advance care planning advisory services to recipients of aged care services. | Descriptive study. Aged care staff and GPs who provide care for older people. All states and territories. | National telephone advisory service (24/7 specialist palliative care advisory service & specialist palliative care advisory service). 21 months. | Findings demonstrated a limited uptake and low call-volume affecting the cost-effectiveness of the advisory service |
|  | Jiang et al (2020) | Assess the feasibility of integrating telehealth-assisted home-based specialist palliative care (TH-SPC) into a rural community setting. | Prospective mixed-methods pilot study. 21 patients (14 received telehealth specialist PC, 7 received standard PC alone). Gippsland, Victoria. | 24-hour telehealth-assisted home-based specialist palliative care. Along with the survival time 5.5 months for intervention group and 6.0 months for standard care group. | The proposed TH-SPC model is feasible and could be integrated successfully into community-based palliative care. Patient and care giver feedback was highly positive, and potential benefits were seen in performance status preservation and health-care resource utilisation. |
